# Molecular Crowder-Induced Structural Transformation of the DNA Dodecamer

**DOI:** 10.1021/acsomega.5c04572

**Published:** 2025-09-01

**Authors:** Neha Mathur, Navin Singh

**Affiliations:** 29794 Birla Institute of Technology and Science, Department of Physics, Vidya Vihar Campus, Pilani, Rajasthan 333031, India

## Abstract

Molecular crowding plays a crucial role in shaping the structural landscape of biomolecules, influencing their stability, interactions, and functional behavior. In vivo, these crowders include biomolecules such as proteins, nucleic acids, and metabolites within the crowded cellular environment, whereas in vitro, they are represented by various organic and inorganic molecules. Understanding how these crowders affect DNA conformation is essential for elucidating their impact on genetic processes. In this study, we explore how molecular crowders influence the structural transformations of a DNA dodecamer sequence using atomistic molecular dynamics simulations. Specifically, we examine the effects of aspartame and polyethylene glycol (PEG-200) as crowding agents. Our findings reveal distinct interaction patterns: while PEG-200 molecules preferentially accumulate near the termini of the DNA, aspartame molecules exhibit a strong affinity for DNA grooves, leading to structural stabilization at lower concentrations and clustering-induced perturbations at higher concentrations. Further, by analyzing key structural descriptors, we elucidate the influence of molecular crowding on DNA organization. These insights contribute to a deeper understanding of how different crowders modulate DNA structure in crowded environments, offering broader implications for biomolecular organization in physiological and biomimetic systems.

## Graphical abstract

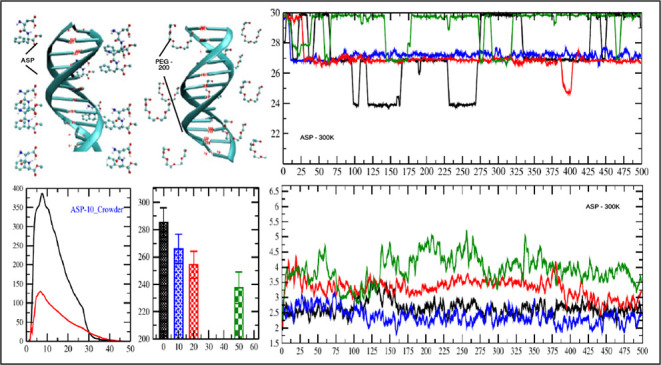

## 1 Introduction

The cellular environment is densely packed, with up to (20–40%) of the cell’s volume consisting of various macromolecules such as nucleic acids, proteins, lipids, and metabolites of various sizes and structures.^1–6^ This dense assembly of molecules is often called macromolecular crowding and can induce changes in the DNA structure, stabilize the configurations or facilitate transitions between different forms.^7–9^ These changes not only influence structural transitions but also affect the efficiency of DNA in carrying out its essential biological functions.^10–12^ Researchers across the globe are working to understand the role of crowders on the intracellular dynamics of DNA molecules.^13–15^ Takahashi et al. investigated the effects of molecular crowding on DNA polymerase reactions using various unnatural DNA templates. They explored how PEG 200 molecules as a crowder influenced the efficiency and preference of DNA polymerization.^16,17^ Punia and Chaudhury investigated the kinetics and thermodynamics of nucleic acid interactions using coarse-grained DNA models. Recently, Punia and Srabanti have studied effect of PEG crowders on DNA capture and translocation through the αHL nanopore.^18^ They studied the conformational properties of DNA duplexes and hairpin structures in the presence of molecular crowders.^10^ Sugimoto and Miyoshi considered different PEGs with molecular weights of 200, 4000, and 8000 and measured the UV absorption and melting temperature of DNA duplexes using fluorescence spectrophotometers.^19–21^ By employing magnetic tweezers, Cheng et al. investigated the impact of monovalent and divalent cations on DNA condensation facilitated by crowders. They demonstrated that PEG of different molecular weights (PEG 600 and PEG 6000) could induce condensation in the presence of *NaCl* and *MgCl*_2_.^22^ Mardoum et al. explored DNA condensation in the presence of different crowder molecules, focusing on how the structural properties of crowders influence DNA conformation and diffusion. They demonstrated that branched and rigid crowders, such as PEG and Ficoll, induce DNA compaction by reducing its conformational volume. In contrast, linear and flexible crowders, such as dextran, promote DNA elongation by increasing its conformational size. Interestingly, their findings revealed that DNA mobility decreases with increasing crowder concentration regardless of the nature of crowders. They also investigated the role of ionic strength on the structural transformation of DNA. They found that the diffusion process and conformational changes in DNA exhibit a complex, nonmonotonic dependence on salt concentration. These effects were attributed to a balance between entropic depletion interactions, which drive compaction or elongation, and electrostatic interactions, modulated by the ionic conditions. Their results provide valuable experimental insights into DNA behavior under crowded conditions. Motivated by their findings, our study employs atomistic simulations to examine the molecular-level interactions between DNA and crowders, specifically PEG and aspartame.^23^ Gao et al. investigated the effects of molecular crowding on the conformation, stability, and ligand interactions of G-quadruplexes. They reviewed significant differences between dilute and crowded environments, emphasizing the need to study *G*4 behavior under physiologically relevant conditions.^24^ Ghosh et al. determined the nearest-neighbor parameters specific to DNA duplex formation under crowded conditions.^25^ They accurately predicted the thermodynamic stability (Δ*H*°, Δ*S°*, and Δ*G*°) and melting temperatures (*T*_m_) of DNA duplexes. To address the intermediate state of DNA, pulled from an end in the presence of crowders, Kumar et al. studied G-quadruplexes (G4s) molecules that display crucial biological functions and are relevant in antitumor and antiviral drug development studies.^7,26^ Using a statistical model, various research groups investigated the melting profile of DNA in the presence of molecular crowders.^27,28^ Recently, Semmeq et al. explored the hydration properties of concentrated aqueous solutions by comparing Polyethylene Glycol (PEG) with Ethylene Glycol (EG). By analyzing solutions of varying concentrations, they discovered distinct microscopic behaviors between PEG and EG.^29^ All these studies (and many more) collectively illustrate the remarkable diversity, complexity, and intriguing nature of the conformations that nucleic acids can assume within DNA-crowder complex systems,^6,12,30^

In this study, we investigated the conformational dynamics of DNA in the presence of two chemically distinct molecular crowders: aspartame, a biologically relevant small molecule^31–33^ and polyethylene glycol (PEG-200), a commonly used synthetic polymer.^16,18,34,35^ While traditional crowders such as PEG are widely employed to model excluded volume effects due to their inert and nonspecific nature, real biological environments also contain small, chemically active molecules that can engage in direct interactions with nucleic acids. Recent reviews, such as Phogat et al., have underscored this limitation, noting that while entropic crowding by inert polymers is well-studied, the role of enthalpic interactions from small, chemically active solutes remains poorly explored. They highlight the need for studies that disentangle volume exclusion from direct, molecule-specific interactions with DNA.^36^

The 12-base pair DNA sequence chosen for this study, 5′–CGCAAATTTGCG–3′ is one of the most thoroughly characterized canonical B-DNA duplexes (PDB **1BNA**, 1.9 Å). Its crystal and NMR data have long served as benchmarks for evaluating force fields, backbone flexibility, and groove hydration, making it a trusted baseline for molecular-crowding studies. Structurally, the duplex combines a centrally located A-tract that narrows and strongly hydrates the minor groove with GC-rich termini that stiffen the helix. Because of this built-in contrast, even small, local crowding effects show up clearly in this duplex, whereas they might be hidden in longer or less defined sequences.

Aspartame, explored here as a biologically relevant molecule crowder, is a widely used artificial sweetener present in many processed foods, carbonated beverages, and pharmaceuticals. Unlike synthetic polymer-based crowders such as PEG, aspartame is a physiologically relevant compound that can interact with biomolecules under cellular conditions. Recent studies suggest that organic molecules, including dietary compounds, may influence DNA stability, hydration, and molecular interactions.^31–33^ Given its amphiphilic nature, aspartame exhibits specific interactions with DNA, such as groove binding and hydrogen bonding, leading to structural stabilization at low concentrations and clustering-induced perturbations at higher concentrations. These properties distinguish it from traditional crowding agents, making it a relevant candidate for understanding nonpolymeric molecular crowding effects in a biologically meaningful context. In contrast, polyethylene glycol (PEG) is a synthetic polymer frequently employed as a molecular crowder in both theoretical and experimental studies. PEG is widely recognized for its biocompatibility, hydrophilicity, and tunable molecular weight range (200–35,000 g/mol). In this study, we selected PEG-200, a low-molecular-weight variant, to contrast its effects with aspartame based on size, structural differences, and physicochemical properties. Unlike aspartame, PEG is largely considered inert and nonspecific in its interactions with DNA, making it a valuable comparison point for assessing how different classes of crowders influence DNA conformational dynamics. Rather than focusing on a single crowder with varying molecular weights, this study examines the effects of two chemically and structurally distinct crowders to elucidate the impact of molecular size, structure, and binding specificity on DNA behavior. By comparing these interactions, we aim to provide insights into how both dietary small molecules and synthetic polymers contribute to molecular crowding effects in biological and biomimetic environments. To investigate these interactions, we employed atomistic molecular dynamics simulations to examine the conformational changes in the DNA sequence (-*CGCAAATTTGCG*-) in the presence of both aspartame and PEG-200 molecules (Figure 1a–f). By analyzing these interactions, we evaluated the impact of different crowders on DNA conformational dynamics. The paper is organized as follows: Section 2 describes the model used in the simulations and details the simulation procedures. Section 3 presents the structural changes observed in the DNA due to the presence of crowders and provides a detailed analysis of the results. Finally, Section 4 summarizes our findings and outlines the conclusions of the study.

**Figure 1:**
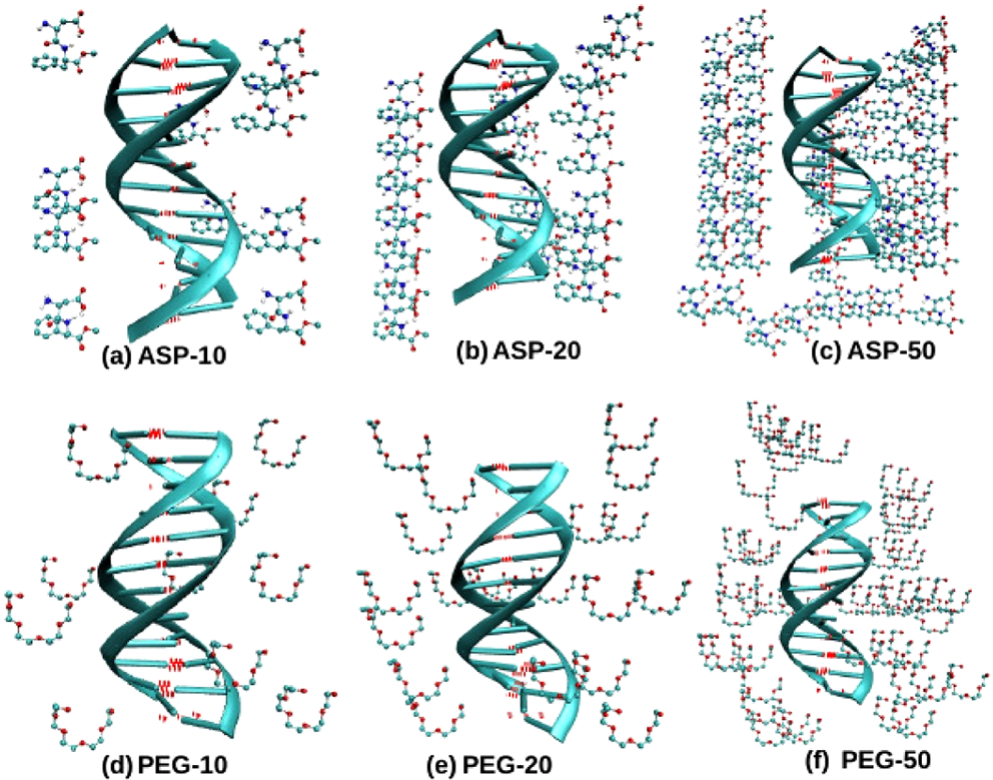
Figures (a–f) present detailed snapshots captured using Visual Molecular Dynamics (VMD), depicting initial DNA configurations under varying concentrations of molecular crowders. Panels (a–c) show DNA surrounded by 10, 20, and 50 aspartame molecules, respectively, illustrating increasing crowding effects. Panels (d–f) replicate this setup with PEG-200 crowders. Images omit water molecules and counterions for clarity; variations in DNA hydrogen bonds are highlighted in red.

## 2 Modeling of the DNA-Crowder System

We used the AMBER22 software package^37^ DNA.bsc1 ^38^ and general amber force field (GAFF)^39^ to study DNA in the presence of molecular crowders. The force field equation is the sum of intra- and intermolecular potential energy terms, including bond stretching *V*_bond_, angle bending *V*_angle_, dihedral torsion *V*_dihedral_, and nonbonded interactions such as van der Waals *V*_vdW_ and electrostatic *V*_electrostatic_ forces.^40^ Mathematically, the total energy of a system can be expressed as follows:

$$V_{\mathrm{total}}=V_{\mathrm{bonded}}+V_{\mathrm{non‐bonded}}$$

For our studies, we use the 3D crystal structure of the DNA dodecamer sequence CGCAAATTTGCG (PDB ID-264D) in the presence of two different kinds of crowders, Aspartame (Figure 1a–c) and Polyethylene Glycol-200 (PEG-200) (Figure 1d–f). Recognizing that biomolecules are randomly distributed in living cells, we varied the location of these crowders around the DNA to replicate cellular conditions in our simulations more accurately. To achieve the desired concentration and distribution of crowders around the DNA, we systematically modified the translation coordinates of the single PEG-200 and Aspartame molecules. By altering the coordinates, we generated multiple copies of each crowder and positioned them randomly around the DNA within the simulation box. Care was taken to ensure that the newly positioned crowders did not overlap with each other or with the DNA molecule. This was done by visually inspecting the positions and making necessary adjustments to the coordinates. To generate the force field parameters of crowders, we use the “Antechamber package” suite to prepare the topology and coordinate files by tleap.^41^ We used the TIP3P model as a reliable and widely accepted representation of water to study various biomolecular processes and interactions in an aqueous environment. The simulations were conducted under salt-free conditions, with only neutralizing counterions (Na+) added to maintain charge neutrality. No additional salt (NaCl or other electrolytes) was introduced in the system to isolate the effects of molecular crowding on DNA structure without the influence of electrostatic screening. We chose box dimensions 55 × 55 × 73 Å with three-dimensional periodic boundary conditions so that the simulated structure always remained inside the box. The box contains 5492 water molecules that were free to move inside and with ions. For electrostatic interaction calculations, we used the Particle Mesh Ewald (PME) method.^42^ We adopted a tolerance criterion of 10^–5^ Å for the direct space summation cutoff. We choose a 10 Å cutoff for electrostatic and nonbonded interactions.

### 2.1 The Minimization Protocol

We put the positional restraints on the solute with a force constant of 500 kcal/mol/Å^2^ and a nonbonded interaction cutoff of 10.0 Å for 5000 cycles, with the first 2500 cycles using the steepest descent method and the remaining cycles using the conjugate gradient method. In the subsequent steps, the system was minimized with progressively reduced restraint forces of 20, 15, 10, and 5 kcal/mol/Å^2^, each with a nonbonded interaction cutoff of 9.0 AA for 5000 cycles. The final step involved removing all restraints, allowing the entire system, including the DNA, to relax freely, with a nonbonded interaction cutoff of 9.0 Å. For the heating phase of our molecular dynamics simulations, we employed the NPT ensemble to maintain the constant number of particles (N), pressure (P), and temperature (T). The system was gradually heated from an initial temperature of 10 K to the target temperature of 300 K over a series of 50,000 MD steps, with a time step of 1 fs. Throughout the heating process, positional restraints were applied to the DNA using a harmonic force constant of 20.0 kcal/mol/Å^2^, restricting the residues to maintain the structural integrity of the solute. Langevin dynamics with a collision frequency of 2.0 was used for temperature control, and the pressure was maintained at 1 atm using isotropic pressure coupling with a coupling constant (taup) of 0.5. The cutoff for nonbonded interactions was set to 9.0 Å. We applied the periodic boundary condition and included the SHAKE constraints on bonds involving hydrogen atoms to ensure efficient sampling and stability. Output data were recorded every 1,000 steps for the trajectory, energy, and restart files, allowing for detailed monitoring of the system’s progress as it reached the desired temperature equilibrium. This careful and controlled heating protocol was crucial for preparing the system for subsequent equilibration and production runs, ensuring a realistic and stable simulation environment. We performed the equilibration phase using the NPT ensemble. Here specifically, we utilized anisotropic pressure coupling with a target pressure set to 1 atm and a pressure coupling constant (taup) of 0.5. The barostat employed in this phase was the isotropic pressure coupling method, while the Particle Mesh Ewald (PME) method was used to ensure accurate and efficient calculation of electrostatic interactions under periodic boundary conditions. We equilibrated the system for two ns to achieve a stable state that accurately reflects the desired temperature, pressure, and structural conditions before commencing the production run. Finally, for the production run of our molecular dynamics simulations, we used the NVT ensemble, maintaining a constant number of particles (*N*), volume (*V*), and temperature (*T*). The system was kept at a constant temperature of 300 K using Langevin dynamics with a collision frequency 2.0. We conducted the simulation for 500 ns. During this phase, no pressure coupling was applied (ntp = 0), ensuring constant volume conditions. The system was initialized from a previously equilibrated state, and the SHAKE algorithm was employed to constrain bonds involving hydrogen atoms, with a tolerance of 10^–6^. For the analysis, throughout the simulation, we calculated the root-mean-square deviation (RMSD) to assess the structural stability and conformational changes of the DNA dodecamer. The radial distribution function (RDF) was computed to analyze the spatial distribution of crowders and ions around the DNA and water shell occupancy to determine the extent of hydration layers surrounding the DNA. All these analyses were performed using the cpptraj tool, and variations in hydrogen bonding were analyzed to understand the dynamic behavior of hydrogen bonds with the nastruct command.^43^

## 3 Results and Discussion

### 3.1 Snapshots of Individual Molecular Dynamics Systems

We first examined the activity of the DNA molecules in the presence of aspartame (ASP) and polyethylene glycol (PEG-200). Initially, to understand the groove-binding properties of aspartame, we designed various systems in which DNA molecules were immersed in a solution containing ten, 20, and 50 aspartame molecules (Figure 1a–c). We adopted computational steps, including minimization, heating, equilibration, and production runs, to analyze the system. Snapshots of the production runs provide insights into the dynamics of the interaction (see Figure 2). With ten ASP crowders, we observed that none of the molecules participate equally in the solution. Approximately six or seven molecules actively participated in interactions, influencing DNA’s structure, whereas the others drifted away (Figure 2a). This selective engagement suggests that ASP crowders tend to interact with DNA; however, this interaction is not uniform among all the molecules present. The variable interactions suggest that while some aspartame crowder molecules stick to the DNA and affect its shape, not all do.

**Figure 2:**
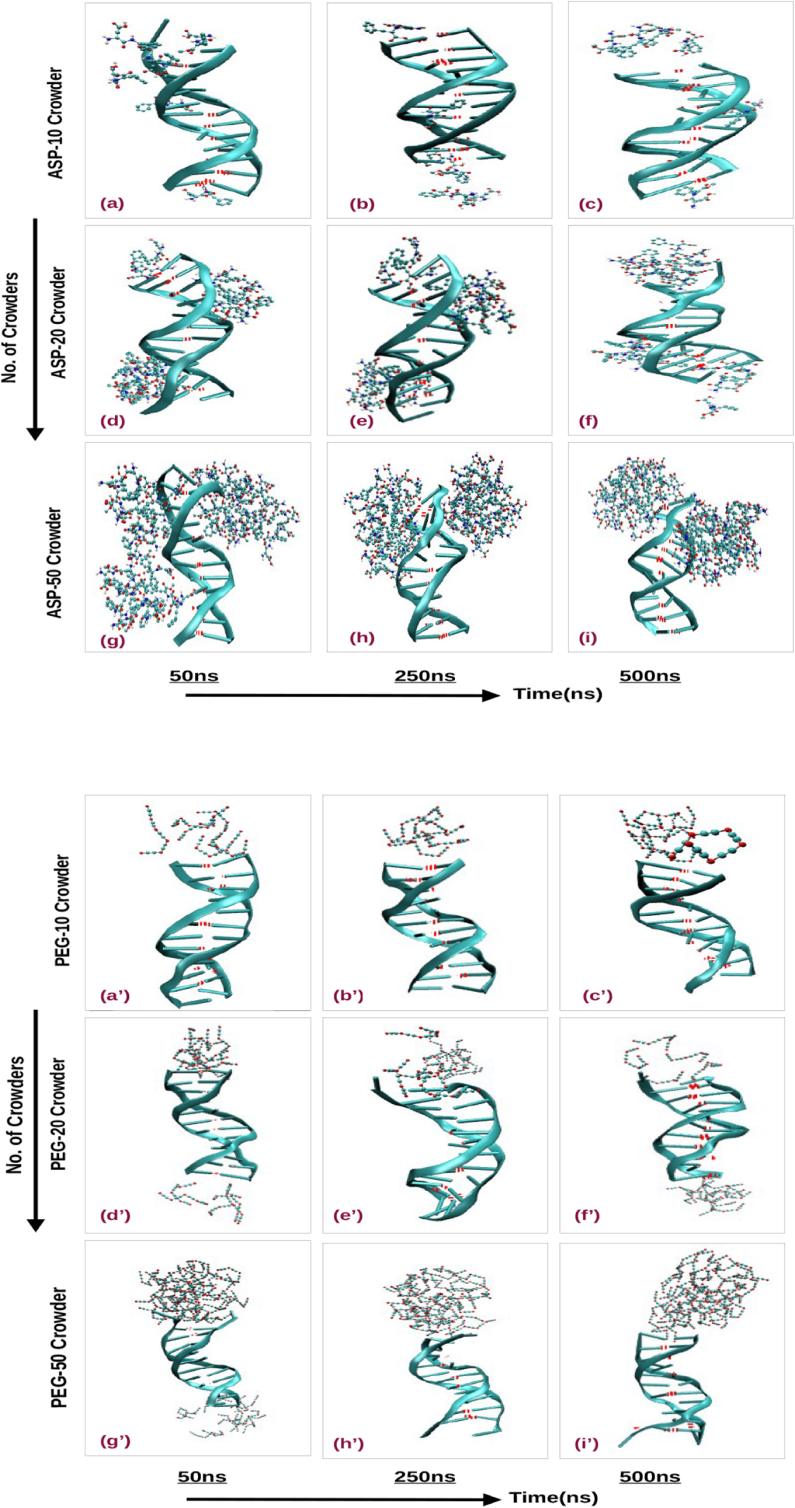
Snapshots depicting DNA-crowder interactions at 50, 250, and 500 ns with increasing concentrations (10, 20, and 50) of aspartame (a–i) and PEG-200 (a’–i’) crowders at 300 K. Images generated from VMD simulations omit water and counterions for clarity, highlighting groove-specific interactions with aspartame and terminal clustering with PEG-200.

Increasing the number of aspartame crowders in our simulations yielded notable effects in the system. These crowders form clusters, initially accumulate within the DNA grooves, and then migrate toward the ends of the DNA molecule. Snapshots with 50 crowders revealed substantial clustering that spanning the entire length of the DNA (Figure 2g–i). This clustering behavior induces significant structural deformations in the DNA, as evidenced by the opening of hydrogen bonds and changes in the overall molecular conformation. In contrast, the PEG-200 crowders exhibited a markedly different interaction pattern. Our simulations indicate that PEG-200 molecules largely avoid direct interaction with the DNA. Even with ten crowders, about half of the PEG-200 molecules stray from the DNA, and no direct interactions with the DNA backbone are observed (Figure 2a’–c’). This trend continued as the number of PEG-200 crowders increased. The crowders formed distinct bunches that preferentially settled at the ends of the DNA rather than within the grooves (Figure 2d’–i’). This behavior contrasts noticeably with aspartame crowders, suggesting that PEG-200 crowders do not affect DNA through groove binding but may still disrupt its structure at the terminal regions. These observations suggest distinct interaction mechanisms for aspartame and PEG-200. Aspartame exhibits a strong affinity for DNA grooves, likely due to its chemical structure, which allows it to form specific interactions with the DNA’s phosphate backbone and base pairs. Depending on the concentration, this interaction results in significant structural changes, including stabilization or distortion. At higher concentrations, aspartame clusters within the grooves, disrupting the hydration shell around the DNA and potentially influencing its structural stability and biological functions, such as gene expression and transcription. In contrast, PEG-200 shows weaker interactions with DNA, primarily localizing at the termini due to its lack of specific affinity for DNA grooves or bases. While PEG-200 exerts minimal effects at low concentrations, its crowding effects become evident at higher concentrations, which induce perturbations at the DNA ends. This observation is consistent with experimental results reported by Sugimoto et al.^44^ where PEG-200 was found to destabilize DNA duplexes by reducing water activity. The accumulation of PEG at terminal regions seen in our simulations supports this hydration-based destabilization mechanism, reinforcing that PEG’s effect is primarily indirect and mediated through solvation shell perturbation rather than specific molecular interactions. These contrasting behaviors underscore the importance of crowder size, chemical composition, and specific affinity in shaping DNA conformational dynamics. The structural characteristics of this DNA sequence play a key role in determining crowder interactions. The narrow minor groove of the A-tract region favors specific binding interactions, explaining aspartame’s preference for groove binding. In contrast, the GC-rich terminal regions reinforce duplex integrity, potentially restricting PEG-200 penetration into the groove and leading to excluded volume effects rather than direct binding.

### 3.2 Calculation of Root Mean Squared Deviation

We calculated the root-mean-square deviation (RMSD) of the DNA to investigate structural changes induced by the presence of crowders. RMSD measures the average displacement of atoms in a simulated structure relative to a reference structure, providing a quantitative assessment of structural deviations. We compared the RMSD of DNA with and without crowders to evaluate the effects of crowder-DNA interactions. The RMSD values, calculated for the DNA backbone atoms (P, O3′, O5′, C3′, C4’, and C5′), are shown in Figure 3a,b. These analyses aimed to explore the impact of crowder number, crowder-DNA interactions, crowder positioning, and crowding saturation on DNA stability and conformation. Simulations of DNA without crowders provided a baseline for its native stability and dynamics under normal conditions.

**Figure 3:**
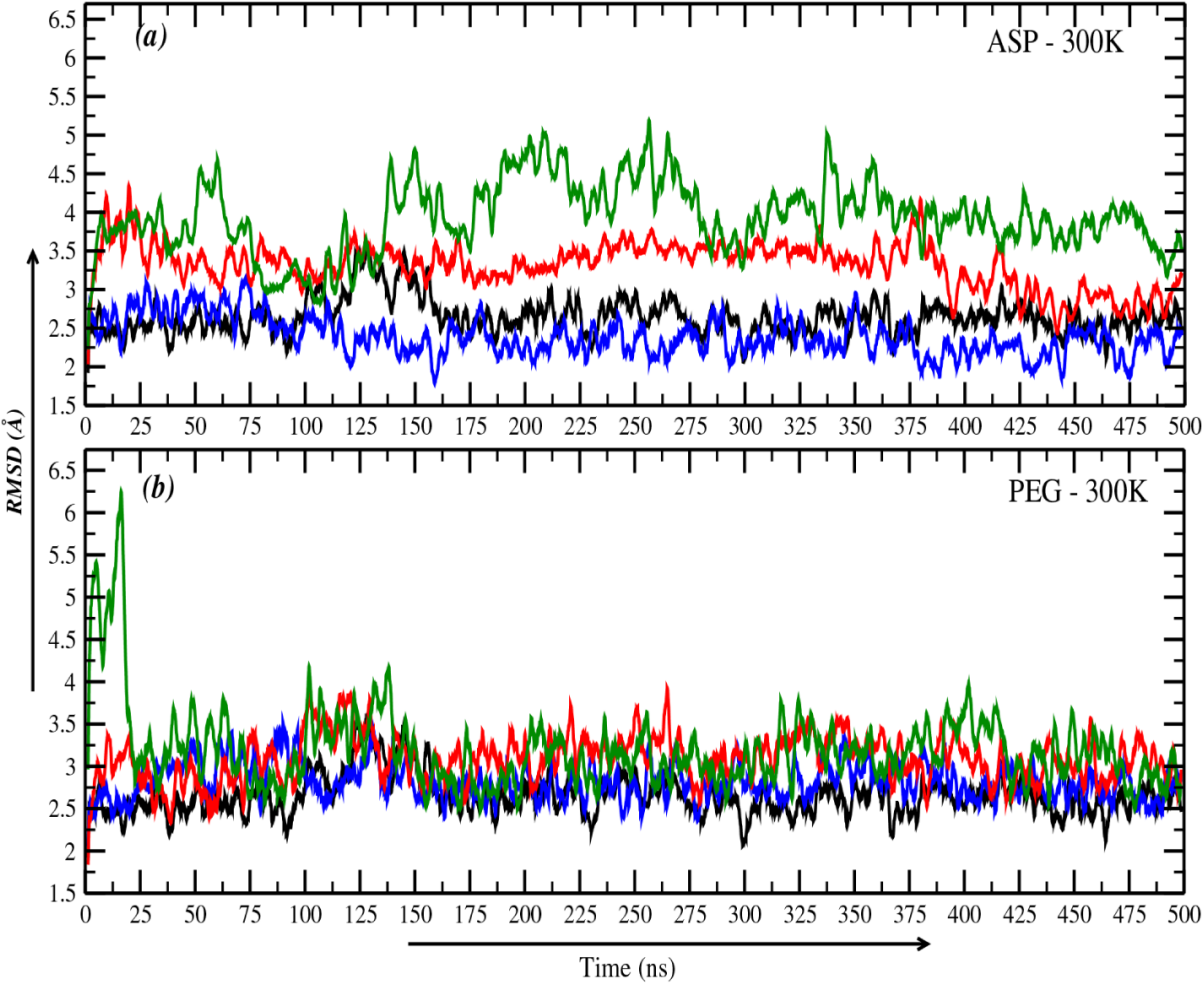
(a) Root mean square deviation (RMSD) of DNA in the presence of aspartame crowders at different concentrations. (b) RMSD of DNA in the presence of PEG-200 crowders at different concentrations. In both panels, the black curve represents the system without crowders (baseline reference), the blue curve corresponds to ten crowders, the red curve to 20 crowders, and the green curve to 50 crowders, showing how crowder concentration influences structural stability.

When ten aspartame crowders were introduced, the RMSD values decreased compared to the baseline. This decrease suggests that aspartame stabilizes the DNA structure at low concentrations. Mechanistically, this stabilization arises from aspartame’s specific affinity for DNA grooves, where it forms hydrogen bonds and electrostatic interactions with the DNA’s phosphate backbone and base pairs. These interactions reduce DNA flexibility, maintaining the structure in a compact conformation closer to its native state. As aspartame crowders increased to 20, the RMSD values rose, indicating a shift toward destabilization. This behavior is likely due to crowding effects, where aspartame molecules cluster within the DNA grooves, disrupting the hydration shell and weakening the hydrogen bonding network. Such clustering introduces perturbations, increasing DNA flexibility and altering its natural conformation. At the highest concentration of 50 aspartame molecules, the RMSD increased even further. This substantial rise reflects the cumulative impact of excessive crowding, where clustering-induced destabilization significantly affects DNA stability and conformation. The introduction of ten PEG-200 crowders had a negligible impact on the RMSD of DNA, with values remaining comparable to the baseline scenario without crowders, which suggests that PEG-200 has weak, nonspecific interactions with DNA, likely due to its inability to bind DNA grooves or interact with the phosphate backbone. At higher concentrations of PEG-200 (20 molecules), there is a minor increase in RMSD, accompanied by fluctuations. These fluctuations reflect transient destabilization due to crowding effects at the DNA termini, where PEG-200 preferentially accumulates. At 50 PEG-200 molecules, the RMSD showed a significant spike (∼6 Å) during the initial 20 ns, followed by stabilization at ∼3 Å. The initial spike is attributed to the crowders disrupting the DNA conformation, while the eventual stabilization suggests that PEG-200 molecules condense and move away from the DNA over time.

### 3.3 Radial Distribution of Crowders and Ions

The radial distribution function (RDF), g­(r) is a valuable measure for analyzing the spatial arrangement and solvation characteristics of molecules in a system. It quantifies the density of particles (either a crowder or an ion) in the vicinity of DNA and provides insight into molecular distribution and interaction patterns. Specifically, *g*(*r*) measures the probability of finding a particle at a distance *r* from a reference particle, although it does not account for directional preferences. In our simulations, *r* represents the center-to-center distance between the reference atoms of DNA (residues 1–24) and the specified atoms of either Na+ ions or crowder molecules (aspartame and PEG-200). In our RDF analysis, the entire crowder molecule was considered as a whole to represent the crowders (aspartame and PEG-200). Specifically, we calculated the radial distribution of the center of mass of the crowder molecules relative to the DNA residues. This approach captures the overall spatial distribution of crowders around DNA, rather than focusing on specific functional groups. While this center-of-mass RDF approach effectively captures global spatial trends, it does have limitations. Because the calculation averages over the entire DNA, including both ends and central regions, any localized clustering such as PEG-200 accumulation at DNA termini or stacking of aspartame molecules along the grooves may be smoothed out in the radial average.

This analysis enables us to understand the distribution of *Na* ^+^ ions and crowders around the DNA molecule. We set the cutoff distance for our RDF analysis at 25 Å, which sufficiently captures the most relevant molecular interactions and solvation effects near the DNA. The results in Figure 4a–c reveal that for 10 and 20 aspartame crowders, the RDF peaks near 7.5 Å, indicating a high probability of crowders binding close to the DNA surface. This behavior is consistent with aspartame’s strong affinity for DNA grooves, driven by its chemical structure, which enables hydrogen bonding and electrostatic interactions with the phosphate backbone. Beyond 7.5 Å, the RDF shows a gradual decline, suggesting that crowders distribute more evenly throughout the solution. When the number of aspartame crowders increases to 50, the probability of finding a crowder near DNA decreases significantly, as reflected in Figure 4c. This decrease arises from the crowding-induced condensation of ASP molecules onto the DNA grooves, which expels hydration water. Such clustering disrupts the natural hydration shell and limits the availability of DNA grooves for further interactions. As a result, aspartame molecules are more likely to stay away from the DNA surface and form clusters, as seen in Figure 2i. Interestingly, the distribution of Na^+^ ions remains largely unaffected by the increased number of aspartame molecules, with the RDF peak consistently located around 6.8 Å. In contrast, the RDF between the center of mass of ASP molecules and the DNA phosphate atoms shows a peak at 3.8 Å (0.38 nm) for ten crowders and 3.1 Å (0.31 nm) for 20 crowders, indicating tighter spatial packing of ASP molecules around the DNA at higher concentrations (Figure 4b). This observation indicates that *Na* ^+^ ions maintain their role in stabilizing the DNA backbone, even under crowded conditions. In contrast, the behavior of PEG-200 crowders, shown in Figure 4d–f, is markedly different from that of aspartame. For all three concentrations (10, 20, and 50 PEG-200 molecules), the RDF for the crowders is lower than that for the ions. The highest RDF peak for PEG-200 occurs around 10.8 Å for 10 and 20 molecules, indicating that PEG-200 crowders are less likely to interact directly with the DNA grooves. At 50 PEG-200 molecules, the RDF distribution becomes flatter, reflecting a more uniform spread of crowders throughout the solution and reduced localization near the DNA. This behavior is consistent with PEG-200s weak, nonspecific interactions with DNA, which result in minimal crowding effects on the DNA surface. The flat RDF at high PEG-200 concentrations corroborates the trends observed in Figure 2a’–i’, where PEG-200 molecules preferentially accumulate away from the DNA. The differences in RDF trends between aspartame and PEG-200 highlight distinct interaction mechanisms. Aspartame’s strong affinity for DNA grooves drives its localization near the DNA surface, leading to pronounced peaks in the RDF at low to moderate concentrations. At high concentrations, crowding effects disrupt this localization and induce clustering, reducing the RDF near the DNA. PEG-200, on the other hand, shows weaker interactions and a preference for uniform distribution, with minimal direct effects on DNA grooves or the surrounding hydration shell.

**Figure 4:**
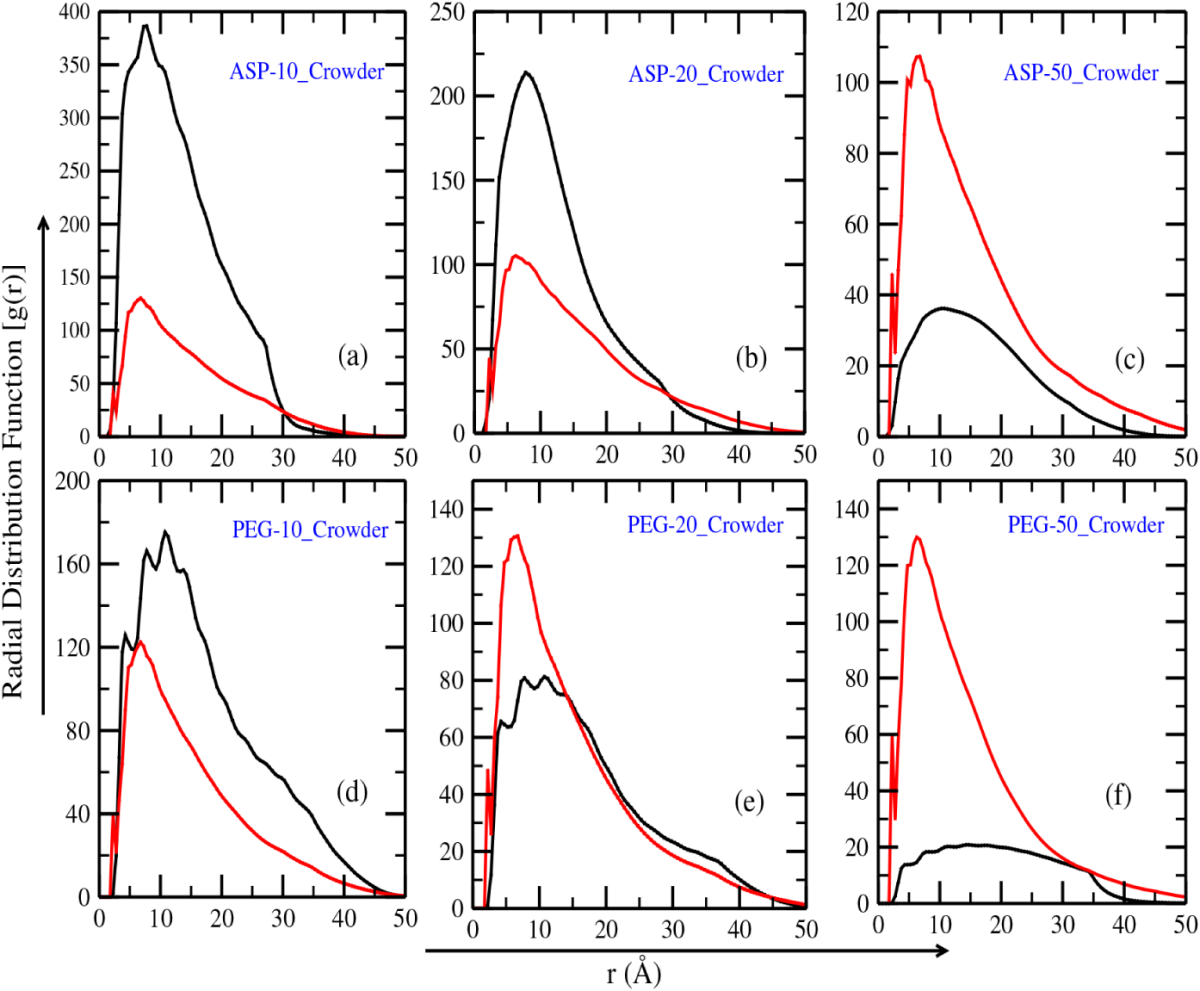
(a–c) depicts the radial distribution functions (RDF) of aspartame crowders and *Na* ^+^ ions around DNA. Similarly, (d–f) illustrates the RDF for PEG-200 crowders and *Na* ^+^ ions, providing insight into their distribution in the surrounding environment. In all plots, the black curve indicates the variation in RDF for the crowders (aspartame and PEG-200), whereas the red curve indicates the RDF for *Na* ^+^ ions.

### 3.4 Water Shell Analysis in the DNA-Crowder Environment

We performed water shell analysis to investigate the solvation properties and behavior of water molecules around the DNA and crowders. This analysis helps to understand the role of the water in stabilizing biomolecular systems and provides a deeper understanding of the stability, dynamics, and molecular interactions of the system. To study how crowders affect DNA hydration, we calculated the number of water molecules within the distance ranges of 3.4–5.0 Å, 3.4–5.5 Å, and 3.4–6.0 Å from the DNA. These distances, measured as surface-to-center values, define successive hydration shells and offer a comprehensive view of solvation behavior. The selected ranges are biologically meaningful, as the first hydration shell typically extends up to approximately 3.4 Å, where solute–water interactions such as hydrogen bonding and electrostatic forces dominate. This immediate layer reflects the direct influence of DNA on nearby water molecules and is essential for understanding solvation properties. Extending the upper limit to 5.5 Å and 6.0 Å allows for the inclusion of dynamically exchanging water molecules in the outer hydration regions, providing a broader perspective on water–DNA interactions.

In Figure 5a–c, we observe that water shell occupancy steadily decreases with increasing numbers of aspartame crowders, from 10 to 50. This behavior suggests that aspartame molecules displace water near the DNA surface, thereby reducing hydration at the DNA-crowder interface. Water occupancy is highest in the absence of crowders, and this difference becomes more pronounced at extended cutoffs, which include more mobile water molecules from the second hydration shell. In contrast, PEG-200 systems (Figure 5d–f) show a less consistent, nonmonotonic trend in water shell occupancy. Although water count is highest without crowders, the occupancy fluctuates across 10, 20, and 50 crowder concentrations. This likely reflects PEG-200s weak and transient interactions with DNA, especially at the termini, which allow more water to remain near the DNA surface. Consequently, PEG-200 causes less disruption to DNA hydration than aspartame. Overall, our findings indicate that aspartame, with its strong groove-binding capacity, significantly disrupts the hydration shell and may alter DNA stability. In contrast, PEG-200s weaker and less specific interactions preserve the hydration environment, supporting a more stable solvation shell. The error bars in each bar diagram represent standard deviations, highlighting the temporal fluctuations in water occupancy during the simulation.

**Figure 5:**
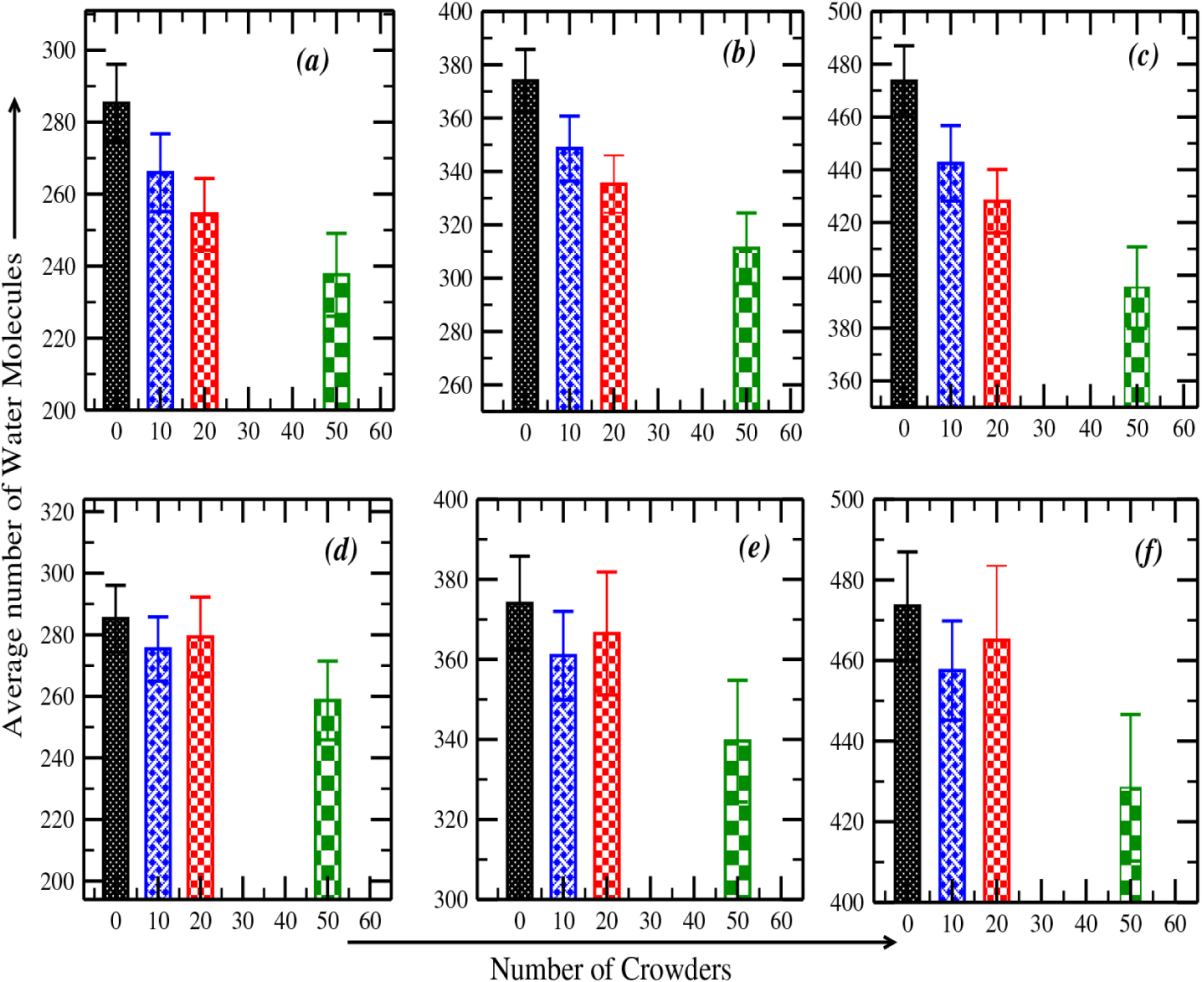
Bar diagrams showing the average number of water molecules within the DNA hydration shell over a 500 ns simulation for varying crowder concentrations. The hydration shell is defined using three distance cutoffs: (a, d) 3.4–5.0 Å, (b, e) 3.4–5.5 Å, and (c, f) 3.4–6.0 Å. Panels (a–c) correspond to aspartame crowders, and (d–f) to PEG-200 crowders. The crowders (0, 10, 20, 50) are plotted along the *X*-axis, and the *Y*-axis represents the average water count within the specified shell. Error bars represent the standard deviation of water occupancy over time. Color coding indicates crowder concentration: black for no crowders, blue for 10, red for 20, and green for 50 crowders.

### 3.5 Analysis of Hydrogen Bonding Variations in Crowded Systems

The study of hydrogen bonding is essential for understanding the formation and stabilization of secondary and tertiary structures of biomolecules. By analyzing H-bonds, we can predict changes in the molecular conformation biological activity and functions of the molecules. For DNA, changes in hydrogen bonding can influence the base pairing, folding/unfolding processes, and solvent effects.

In the absence of crowders, the number of hydrogen bonds in DNA fluctuates between 24.0–30.0 due to thermal energy and interactions with water molecules. These fluctuations represent the natural dynamics of hydrogen bonding under normal conditions. This baseline serves as a reference to evaluate the impact of crowders on hydrogen bonding in DNA. When we introduced 10 and 20 aspartame molecules into the system (Figure 6a), we observed reduced fluctuations in the number of hydrogen bonds, stabilizing them around 27. This stabilization suggests that aspartame interacts specifically with DNA grooves, promoting consistent hydrogen bonding. At lower concentrations, aspartame crowders do not significantly displace water molecules from the grooves, allowing water to assist in maintaining the DNA’s hydrogen bond network. As the number of aspartame crowders increased to 50, we observed larger clusters of crowders forming near the DNA. These clusters displaced more water molecules from the DNA grooves, but their presence along the DNA chain provided additional stabilization through direct interactions with the DNA structure. Interestingly, the hydrogen bond count increased slightly, fluctuating between 27 and 30, indicating enhanced stability. This behavior highlights the dual role of aspartame: at lower concentrations, stabilization arises from specific groove interactions, while at higher concentrations, stabilization results from crowder clustering along the DNA chain, reducing flexibility and maintaining hydrogen bonds. The behavior of PEG-200 crowders (Figure 6b) differs significantly from that of aspartame. At low concentrations (10 PEG-200 molecules), crowders primarily accumulate at the DNA termini, where hydrogen bonds are inherently weaker. Consequently, there is little to no impact on the overall hydrogen bonding network. However, as the number of PEG-200 molecules increases to 20 and 50, the crowders form clusters at the DNA ends, leading to osmotic pressure and localized destabilization. This clustering exerts mechanical forces on the termini, causing some hydrogen bonds to break and destabilizing the double-stranded DNA structure. The reduction in hydrogen bonds, fluctuating between 24 and 27, reflects this destabilization. Unlike aspartame, PEG-200 crowders do not interact specifically with DNA grooves and exhibit weaker binding affinity. Their primary influence is localized at the DNA termini, where clustering forces cause partial opening of the double-stranded DNA. This behavior underscores the nonspecific, size-dependent effects of PEG-200 on DNA hydrogen bonding. Hence, the observed differences in hydrogen bonding behavior between aspartame and PEG-200 are driven by their distinct interaction mechanisms, where aspartame acts as a groove binder. At the same time, PEG-200 induces clustering-driven destabilization at the DNA ends, breaking hydrogen bonds and partially unwinding the DNA.

**Figure 6:**
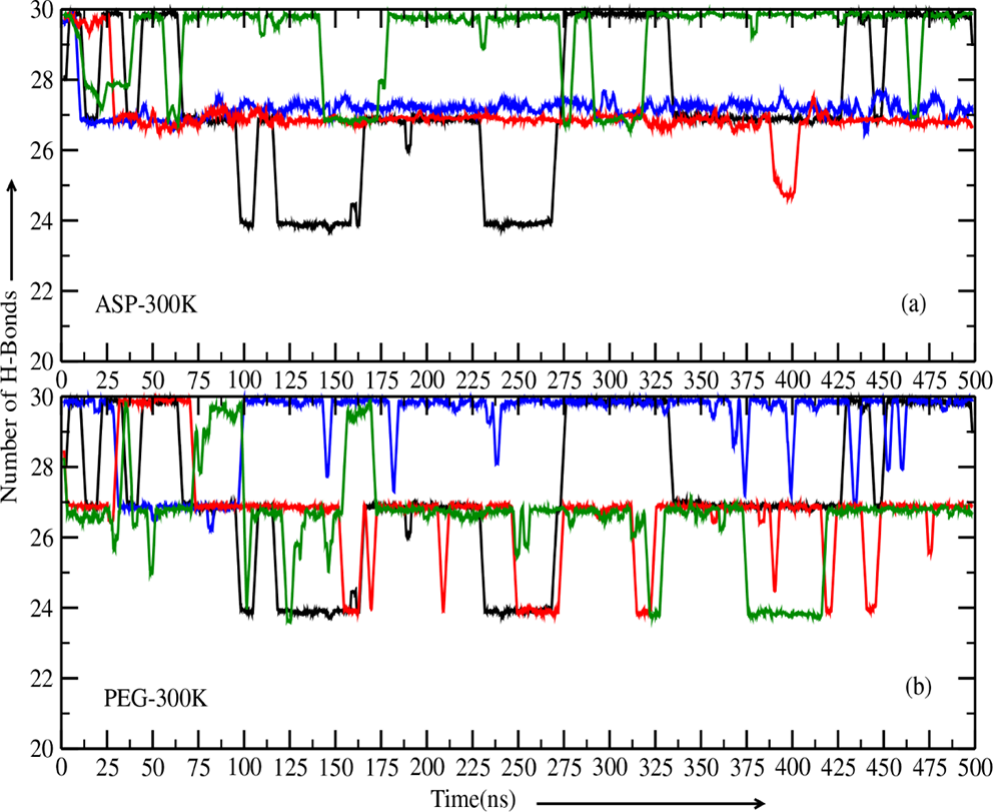
(a) The variation in the number of hydrogen bonds for DNA surrounded by aspartame crowders. (b) The variation in the number of hydrogen bonds for DNA surrounded with PEG-200 crowders. The black line represents the condition with no crowders present, serving as a baseline for comparison. The blue, red, and green lines depict the scenarios with 10, 20, and 50 crowders, respectively, indicating the presence and concentration of different crowders and their influence on hydrogen bonding in DNA. We also employed a running average of 300 to smooth out short-term fluctuations and highlighted the significant trends using the xmgrace tool.

## 4 Conclusion

We present a comprehensive analysis of crowder-DNA interactions, focusing on the effects of polyethylene glycol (PEG-200) and aspartame on DNA stability, hydration, and conformational dynamics. We highlight the interplay between crowders and DNA under crowded conditions by integrating multiple analyses, including simulation snapshots, RMSD, radial distribution function (RDF), water shell occupancy, and hydrogen bonding variations. Our findings reveal distinct interaction mechanisms for aspartame and PEG-200. Aspartame exhibits a strong affinity for DNA grooves, forming specific interactions with the phosphate backbone and base pairs. These interactions result in structural stabilization at low concentrations but lead to clustering-induced disruption of the hydration shell and destabilization at higher concentrations. In contrast, PEG-200 interacts weakly and nonspecifically, localizing primarily at the DNA termini. Its effects are minimal at low concentrations but induce localized perturbations at higher concentrations due to clustering effects. PEG-200 was selected for this study because of its well-documented effects on biomolecules and relatively small molecular size, allowing for a detailed exploration of localized interactions. Its size offers a balance between inducing macromolecular crowding and compatibility with simulation conditions, making it ideal for comparison with aspartame. Although other molecular weights of PEG could have been used, PEG-200 provides insight into how smaller synthetic molecules influence DNA structure. Aspartame was included to bring biological relevance and novelty to the study. As a dietary molecule with growing concerns about its potential genotoxic effects, aspartame allows studying DNA interactions under conditions that mimic real-world biological environments. The combination of PEG-200 and aspartame directly compares synthetic and biologically relevant crowders, highlighting their distinct effects under similar conditions. The RMSD analysis illustrates how crowder concentration impacts DNA stability and flexibility. At low concentrations, aspartame stabilizes DNA through specific interactions such as hydrogen bonding and electrostatic forces. However, higher concentrations result in clustering, which disrupts the hydration shell and weakens hydrogen bonding, leading to increased DNA flexibility and structural perturbations. In contrast, PEG-200 shows negligible effects at low concentrations but induces localized destabilization at the DNA termini at higher concentrations. Over time, these perturbations stabilize as PEG-200 crowders condense and move away from the DNA, highlighting their limited impact on overall DNA conformation. To further explore the spatial distribution of crowders, we analyzed the radial distribution function (RDF). Aspartame molecules strongly prefer DNA grooves, accumulating within a radius of 7.5 Å. Increasing aspartame concentration decreases RDF values near the DNA while increasing the RDF for ions, reflecting the competitive displacement of crowders and ions. PEG-200, by contrast, displays lower RDF values, indicating weak and diffuse interactions with DNA. This difference underscores aspartame’s extensive interaction along the DNA chain compared to PEG-200s localized effects. Water shell analysis reveals how crowders displace water molecules from the vicinity of DNA, influencing its structural stability. Increasing aspartame concentration leads to significant disruption of the hydration shell, particularly within DNA grooves, due to its strong, specific interactions. In contrast, PEG-200s weaker, nonspecific interactions result in a less disrupted hydration environment, primarily through excluded volume effects. These findings confirm that crowder-induced changes in hydration play a crucial role in modulating DNA stability, highlighting the differential impact of specific versus nonspecific molecular crowding on nucleic acid behavior.^45^ Finally, hydrogen bonding analysis highlights the distinct mechanisms of stabilization and destabilization for the two crowders. Aspartame stabilizes hydrogen bonds along the DNA chain by interacting with grooves, and at higher concentrations, clustering enhances this stabilization. In contrast, PEG-200 disrupts hydrogen bonds at the DNA termini, where clustering exerts mechanical forces, leading to localized destabilization and partial unwinding of the DNA. By integrating these findings, we provide a unified perspective on how crowders modulate DNA behavior. Aspartame’s strong groove-binding interactions and clustering behavior at higher concentrations suggest potential biological implications, including gene expression and transcription interference. This is particularly concerning given recent studies linking aspartame to potential genotoxic effects and cancer risk.^32,33,46,47^

While aspartame is not a classical macromolecular crowder in the entropic sense, its clustering behavior induces crowding-like effects such as hydration shell disruption and local compaction. This aligns with current perspectives that classify certain small molecules and osmolytes as chemically active crowders, acting via both enthalpic and entropic mechanisms. Although high intracellular concentrations of aspartame may not be physiologically typical, our study provides insight into how elevated exposure such as through excessive dietary intake may influence DNA structure. In this context, aspartame serves as a valuable proxy for understanding how small, biologically relevant molecules modulate nucleic acid conformation and hydration through combined binding and crowding effects. In contrast, PEG-200, with its weaker and less specific interactions, serves as a less intrusive crowder, making it a valuable model for studying macromolecular crowding effects in biomimetic systems. Altogether, our findings show that a small, chemically active crowder like aspartame reshapes DNA in ways an inert polymer such as PEG-200 cannot. Crowding does more than squeeze space it rearranges the local water–ion network, so that changes in crowder size, chemistry, and concentration can make a duplex compact, melt, or even switch into exotic forms like G-quadruplexes.^48^ By directly comparing a synthetic polymer with a biologically relevant molecule, we reveal how crowder chemistry and binding propensity govern DNA stability, hydration, and architecture under realistic, well-controlled conditions. This unified perspective is crucial for in-cell modeling, nanopore sensing, and structure-guided drug discovery, where selecting crowders that truly mimic the cellular environment makes all the difference.

## Supplementary Material



## Data Availability

All data supporting the findings of this study are provided within the article and its Supporting Information files.

## Notes

Radius of gyration (RoG) plots for DNA backbone atoms under varying concentrations of aspartame and PEG-200 crowders (Figure S1); end-to-end distance profiles between terminal residues of DNA duplexes in the presence of aspartame and PEG-200 crowders (Figure S2) (ZIP)
