# Supplementary figures and images for: Molecular Crowder-Induced Structural Transformation of the DNA Dodecamer
Source: ACS Omega. 2025 Sep 1;10(36):41425–35. doi: 10.1021/acsomega.5c04572 (PMC12444521; doi:10.1021/acsomega.5c04572)

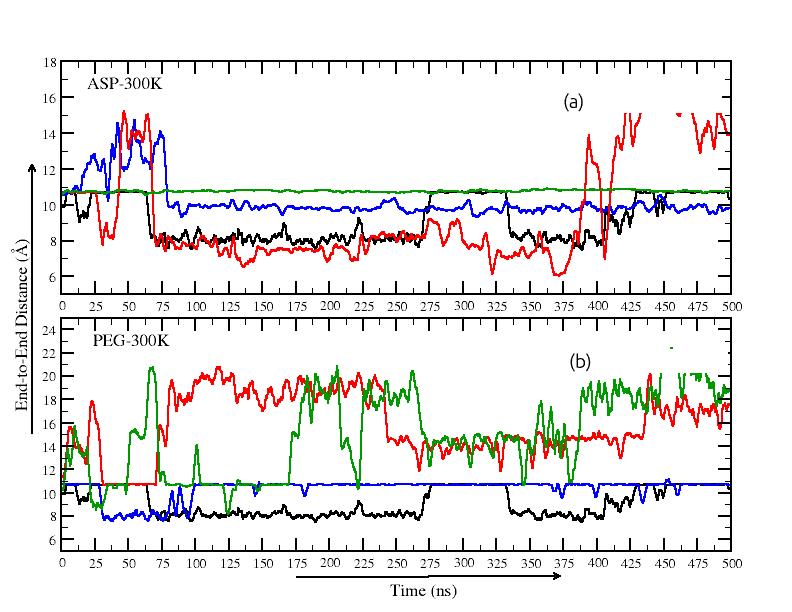

Supplement: Supplementary file 1 [file ao5c04572_si_001.zip › end-end.png]

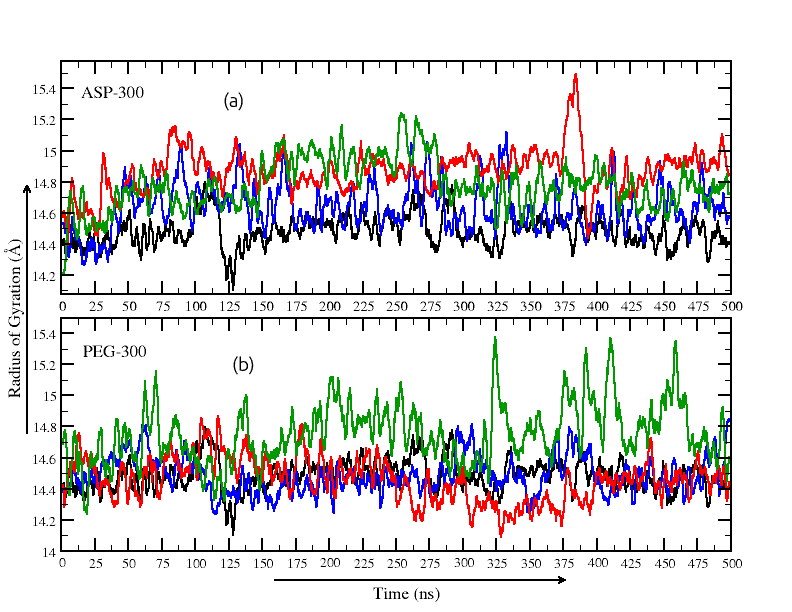

Supplement: Supplementary file 1 [file ao5c04572_si_001.zip › rog.png]
